# Pulsed Electric Field Induces Significant Changes in the Metabolome of *Fusarium* Species and Decreases Their Viability and Toxigenicity

**DOI:** 10.3390/toxins17010033

**Published:** 2025-01-11

**Authors:** Adam Behner, Jana Palicova, Anna-Hirt Tobolkova, Nela Prusova, Milena Stranska

**Affiliations:** 1 Department of Food Analysis and Nutrition, University of Chemistry and Technology, Prague, Technicka 3, 166 28 Prague, Czech Republic; adam.behner@vscht.cz (A.B.); anna.tobolkova@gmail.com (A.-H.T.); nela.prusova@vscht.cz (N.P.); 2 Crop Research Institute in Prague, Drnovska 507/73, 161 00 Prague, Czech Republic; palicova@vurv.cz

**Keywords:** *Fusarium*, food pathogens, pulsed electric field, metabolomic fingerprinting, UHPLC-HRMS/MS, biomarkers, mycotoxins, spore viability

## Abstract

*Fusarium* fungi are widespread pathogens of food crops, primarily associated with the formation of mycotoxins. Therefore, effective mitigation strategies for these toxicogenic microorganisms are required. In this study, the potential of pulsed electric field (PEF) as an advanced technology of increasing use in the food processing industry was investigated to minimize the viability of *Fusarium* pathogens and to characterize the PEF-induced changes at the metabolomic level. Spores of four *Fusarium* species (*Fusarium culmorum*, *Fusarium graminearum*, *Fusarium poae*, and *Fusarium sporotrichioides*) were treated with PEF and cultured on potato dextrose agar (PDA) plates. The viability of the *Fusarium* species was assessed by counting the colony-forming units, and changes in the mycotoxin content and metabolomic fingerprints were evaluated by using UHPLC-HRMS/MS instrumental analysis. For metabolomic data processing and compound identification, the MS-DIAL (v. 4.80)–MS-CleanR–MS-Finder (v. 3.52) software platform was used. As we found out, both fungal viability and the ability to produce mycotoxins significantly decreased after the PEF treatment for all of the species tested. The metabolomes of the treated and untreated fungi showed statistically significant differences, and PEF-associated biomarkers from the classes oxidized fatty acid derivatives, cyclic hexapeptides, macrolides, pyranocoumarins, carbazoles, and guanidines were identified.

## 1. Introduction

Microscopic filamentous fungi of the *Fusarium* genus are among the most widespread plant pathogens [1]. These pathogens are responsible for many plant diseases, including seedling blights [2], root rots [3], and Fusarium head blight (FHB) [4], and can cause major losses in crop yield and quality, particularly when affecting cereal crops. Crop contamination is also accompanied by the presence of toxic secondary metabolites, mycotoxins, which represent a serious health risk to farm animals and humans [5]. For these reasons, together with the fact that climate change scenarios predict further increases in crop contamination [6], control, prevention, and decontamination strategies are urgently needed.

Prevention strategies involve adopting the best agricultural, manufacturing, and hygiene practices to mitigate the growth and spread of fungi, followed by mycotoxin synthesis [7]. The decontamination approach is based on chemical, biological, or physical methods, where the physical methods include technologies such as cold plasma [8], pulsed light [9], ozone treatment [10], ultrasound [11], and pulsed electric field (PEF) [12]. Of these, PEF technology, utilizing the application of short and intensive electric pulses on microbial pathogens, appears to be the most promising from the viewpoint of the mitigation of pathogenic fungi and mycotoxin reduction, with minimal effects on food taste, color, and nutrient content [13].

PEF technology has been used in the food industry for decades. Its antimicrobial effect is based on the fact that the high-potential electrical pulses with defined parameters in the environment of a conductive food matrix lead to the permeabilization of microorganism cell membranes and result in cell death [12,14]. Until now, it has been used mainly for the preservation of milk, eggs, juices, and other liquid foods, and its efficiency has been demonstrated mainly for yeasts and bacteria [15,16,17]. Although some studies indicating the potential of PEF to eliminate microscopic fungi have been published, this information is rather limited. Zhong et al. [18] demonstrated this phenomenon in *Fusarium* mycelium cells during PEF treatment of nutrient solutions. Evrendilek et al. [12] observed a significant reduction in pathogenic *F. graminearum* after PEF treatment of wheat, barley, parsley, onion, lettuce, tomato, and garden rocket seeds. A recent study by Palicova et al. [19] investigated the ability of *Fusarium* spores to survive PEF treatment with different intensities.

From a practical point of view, PEF technology has great potential in food technologies using “wet” technological steps, as it needs an electric current conductor. This is why intensive research on its application in malting technology for reducing mycotoxins was performed recently [20]. The aim was to apply PEF to barley containing *Fusarium* and *Alternaria* mycotoxins and reduce their content by degradation and increased extraction to the steeping water [20]. However, to the best of our knowledge, the direct influence of PEF on *Fusarium* fungus spores, as well as the impact of PEF on their metabolome, has not been investigated.

The present paper provides new information about the viability of *Fusarium* spores, the toxigenicity of the developed fungi, and the metabolomic changes during fungal maturation induced by the PEF treatment of spores of four well-known *Fusarium* species (*F. culmorum* (FC), *F. graminearum* (FG), *F. poae* (FP), and *F. sporotrichioides* (FS)). This pilot study provides important information exploitable for combatting this important food pathogen.

## 2. Results

### 2.1. UHPLC–HRMS/MS Method Optimization

One of the most important steps during the development of the UHPLC-HRMS/MS fingerprinting method was the optimization of the extraction procedure. In general, the extraction efficiency must be very broad and cover a wide range of metabolite polarities to avoid discrimination between some important biomarkers. The optimization of the extraction included testing four extraction solvents/solvent mixtures representing a different range of polarities, in particular methanol/propan-2-ol (50:50, *v*/*v*), methanol, methanol/water (50:50, *v*/*v*), and water. The final choice of extraction solvent depended on the absolute numbers of polar (eluting to 6 min RT), middle-polar (eluting from 6 to 12 min RT), and less polar metabolites (eluting between 12 and 19 min RT) to ensure a sufficient number of metabolites with different physicochemical properties. Accordingly, the best results were found for methanol/propan-2-ol, with an absolute number of 18,227 features (13,008 and 5219 features in positive (ESI+) and negative (ESI-) ionization mode). Nevertheless, according to Table 1, a methanol extract with an absolute number of features of 17,583 (12,109 and 5474 features in ESI+/ESI−) was more representative in the polar region and therefore chosen as the best option. The TIC (total ion current) records of the UHPLC-HRMS/MS fingerprints of the PEF-treated and control samples (FC, FG, FP, and FS) are shown in Figures S1–S4 of the Supplementary Materials. Although, at first sight, the TIC records of the PEF-treated and control samples were very similar, subsequent chemometric analysis identified clear differences. 

### 2.2. Chemometric Analysis

**Figure 1:**
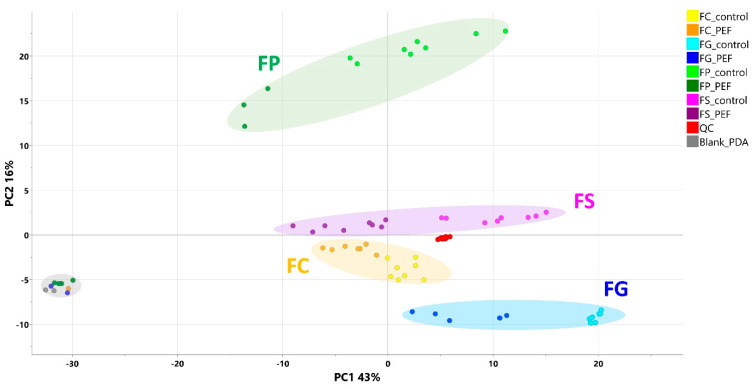
PCA (score scatter plot) including all samples (FC—*F. culmorum*; FG—*F. graminearum*; FP—*F. poae*; FS—*F. sporotrichioides*) colored according to the particular experimental conditions. Excluded PEF-treated samples are grouped with the “PDA blank” samples represented by gray dots in the left part of the plot.

**Figure 2:**
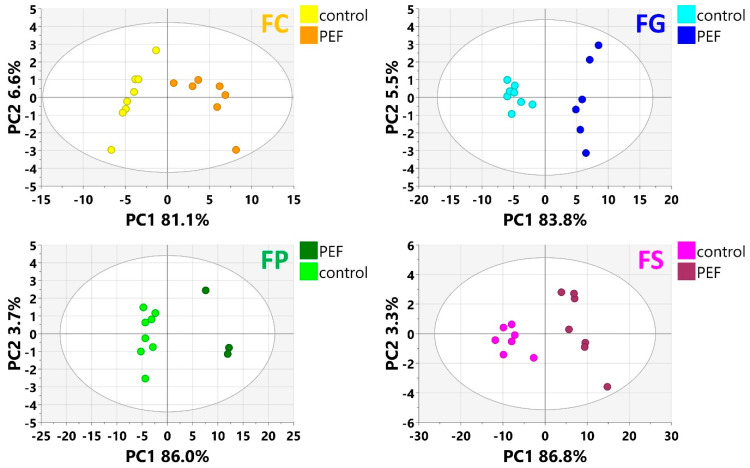
PCA (score scatter plots) of each *Fusarium* species dataset (FC—*F. culmorum*; FG—*F. graminearum*; FP—*F. poae*; FS—*F. sporotrichioides*) colored according to the particular experimental conditions.

The workflow pipeline, including the MS-DIAL (v. 4.80)–MS-CleanR–MS-FINDER (v. 3.52) software platform described in Section 5.6, was used to process the raw data files generated by the UHPLC-HRMS/MS analysis. In the first step, all the data were processed together by PCA to provide a first view of the differences between the metabolomes of samples from various groups. Figure 1 presents the PCA score scatter plot, where samples are colored according to *Fusarium* species and PEF-treated/untreated control groups. As seen from this figure, some PEF-treated samples were grouped together with the “PDA blank” samples. After a detailed check of the photos of the PDA plates, we found that on these plates, *Fusarium* mycelia were totally absent (in other words, PEF deprived the spores of their ability to germinate and form mycelia), so these samples were excluded from subsequent data analysis. In the next step, the data were processed as binary models for treated/non-treated variants for each *Fusarium* species, as described in Section 5.7. Then, for each species, PCAs were performed again, and as can be seen from Figure 2, the cluster separation was even better, promising good potential for follow-up supervised mathematical–statistical models and statistically significant marker generation.

To determine which features were most responsible for the clear clustering of samples between the PEF-treated and control samples in all datasets, supervised OPLS-DA models were built (see Figure S5). The values of all the quality parameters for all models are summarized in Table S1. The quality of all classification models was excellent according to the model classification by Triba et al. [21], the Q2 intercept values from the permutation tests (determined after 100 permutations) were below 0, and all models classified all samples into the known classes 100% correctly.

**Figure 3:**
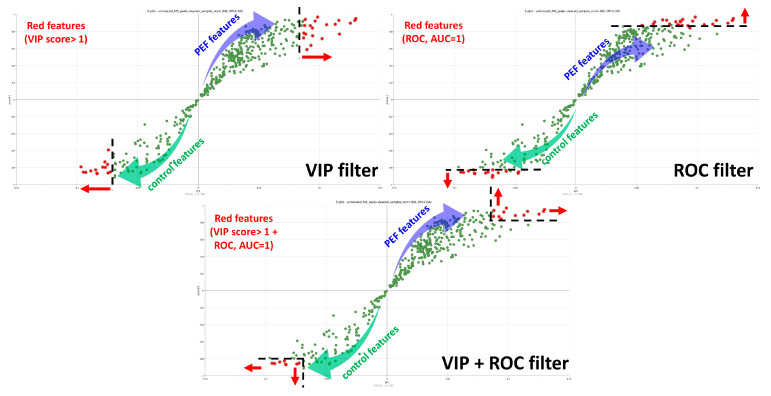
Selection of statistically significant features using VIP + ROC filters illustrated in the OPLS-DA S-plot.

The most significant features responsible for the clustering of the PEF-treated and control samples, referring to biomarkers of metabolic changes in fungal metabolism after PEF treatment, were selected using VIP plots (VIP score > 1) combined with ROC analysis (AUC = 1). The simultaneous application of these two independent orthogonal statistical filters enabled the selection of a relatively low number of biomarkers, which were, however, very powerful and unique, which is essential for effective data interpretation. The selection of statistically significant features from the S-plot using a combination of VIP and ROC filters is illustrated in Figure 3.

### 2.3. PEF Biomarkers

Through this procedure, two groups of statistically significant features were selected for each dataset (where the PEF-related features represented biomarkers up-regulated after PEF treatment, whereas the control ones represented biomarkers down-regulated in reaction to PEF). In particular, 19 biomarkers related to PEF treatment (4 up-regulated, 15 down-regulated) were selected for FC, 26 biomarkers (21 up-regulated, 5 down-regulated) for FG, 40 biomarkers (24 up-regulated, 16 down-regulated) for FP, and 60 biomarkers (53 up-regulated, 7 down-regulated) for FS. All these biomarkers are summarized in the Candidates of PEF-related biomarkers and Candidates of control-related biomarkers .xlsx supplementary files, together with the mass spectrometric characteristics and the VIP/ROC selection parameters.

**Figure 4:**
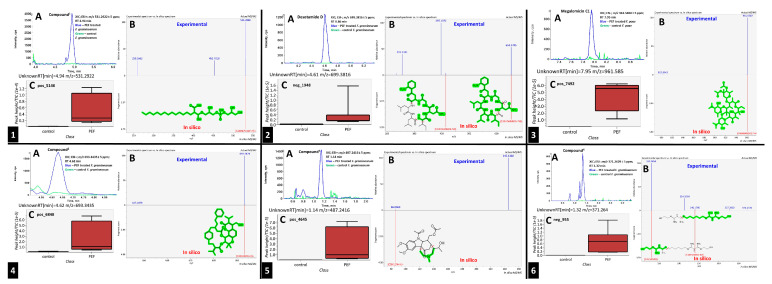
The extracted ion chromatograms (XICs) (**A**), match of experimental vs. in silico MS/MS spectra with chemical structures of fragments colored green (**B**), and boxplots before statistical analysis (**C**) of unique PEF-related biomarkers Compound^1^ (**1**), Desotamide D (**2**), Megalomicin C1 (**3**), Compound^2^ (**4**), Compound^3^ (**5**), and Compound^4^ (**6**). Tentative identifications and intensity trends of these biomarkers are presented in the FG and FP datasets. ^1^ 2-((3-(((2,3-dihydroxypropoxy)(hydroxy)phosphoryl)oxy)-2-hydroxypropoxy)(hydroxy)methyl)hexadecanoic acid. ^2^ 13-(hydroxymethyl)-19,19-dimethyl-3-(2-phenylethyl)-12-(propan-2-yl)-23-(propan-2-ylidene)-6,10,18,21-tetraoxapentacyclo[24.2.2.0^7^,^20^.0^8^,^17^.0^9^,^14^]triaconta-1(28),8,12,14,16,26,29-heptaene-5,11,22-trione. ^3^ [8-acetyl-12-(1-hydroxyethyl)-4,5-dimethoxy-14-methyl-17-oxo-8,14-diazatetracyclo[9.5.2.0¹,⁹.0²,⁷]octadeca-2(7),3,5,12-tetraen-10-yl]methyl acetate. ^4^ 1-[N′-[6-[[amino-[[N′-(2-hydroxyethyl)amidino]amino]methylene]amino]hexyl]amidino]-2-(2-hydroxyethyl)guanidine.

In the second step, biomarkers overlaying between the *Fusarium* species were investigated in more detail. Table 2 summarizes the PEF-related (up-regulated) biomarkers occurring exclusively in the PEF-related samples and in more than one *Fusarium* species. As can be seen from this table, one of the most commonly occurring PEF-related biomarker was 2-((3-(((2,3-dihydroxypropoxy)(hydroxy)phosphoryl)oxy)-2-hydroxypropoxy)(hydroxy)methyl)hexadecanoic acid, which was present in the mycelia of FG, FP, and FS. The other biomarkers belonged to the chemical classes cyclic hexapeptides, derivatives of N-acyl-alpha amino acids, pyranocoumarins, carbazoles, guanidines, and macrolides (see Table 2). The intensity trends of these biomarkers, shown as intensity boxplots, together with the raw mass spectrometric data, are shown in Figure 4. It is worth noting that this final check of the biomarker signals in the raw UHPLC-HRMS/MS data (not influenced by any kind of statistical intervention) is one of the most important steps confirming the diagnostic strength of a particular feature.

In the case of the control-related (down-regulated) biomarkers, the overlays between each *Fusarium* species were observed at the level of chemical ontology only (no metabolites that were structurally the same were found in the different *Fusarium* species, but some of them were of the same chemical groups, as classified by MS-FINDER software (v. 3.52)). These overlays are summarized in Table S2 and include triterpenoids, N-acyl-alpha amino acids and derivatives, and oligopeptides.

### 2.4. Influence of PEF on Fungal Viability and Mycotoxin Production

In addition to metabolomic analysis, the effect of PEF on the spore viability and mycotoxin production of *Fusarium* fungi was investigated. An important fact is that the viability of all four *Fusarium* species (FC, FG, FP, and FS) showed a statistically significant decrease after PEF treatment of the spores when compared to the control (see Table S3). However, the extent of this response to PEF was not the same and varied between the species. FP spores were the most sensitive and showed a strong reduction in viability (9.4% of surviving spores). On the other hand, FC spores were shown to be the most resistant (53.4% of surviving spores).

**Figure 5:**
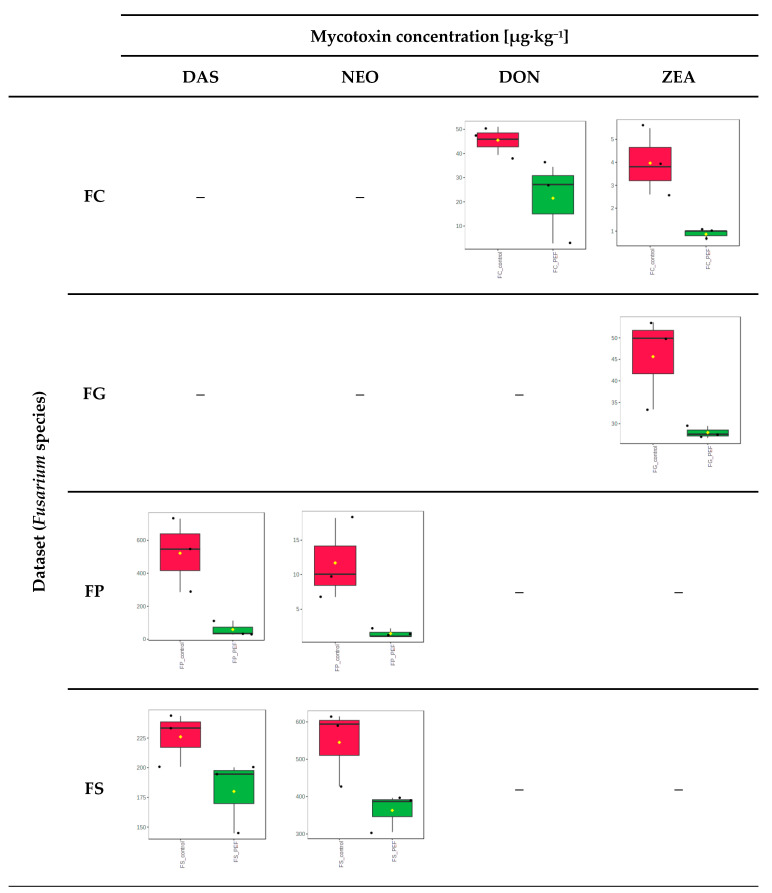
Boxplots demonstrating the distribution of mycotoxin (diacetoxyscirpenol (DAS), deoxynivalenol (DON), neosolaniol (NEO), and zearalenone (ZEA)) abundance in PDA plates with fungi (FC—*F. culmorum*; FG—*F. graminearum*; FP—*F. poae*; FS—*F. sporotrichioides*) only with significant decreases after PEF treatment. Significance was statistically tested using the Wilcoxon rank-sum test (*p*-value < 0.1). Red boxplots represent control samples, and green boxplots represent PEF-treated samples. The black dots in the boxplot represent each sample and the yellow square represents the average.

As concerns the influence of PEF on the potential of fungi to form mycotoxins, the UHPLC-HRMS/MS analyses performed on three biological replicates of all variants (*Fusarium* species and PEF-treated vs. control samples) revealed interesting facts. Of all 22 *Fusarium* mycotoxins included in our method, altogether, 9 toxins were semi-quantified in PDA cultivates of our FG, FC, FS, and FP strains (see Table S4), with type B trichothecenes, including deoxynivalenol (DON) and its acetylated derivatives, as the main metabolites produced by FC, zearalenone (ZEA) as the main mycotoxin produced by FG, and type A trichothecenes, including T2 toxin, HT2 toxin, diacetoxyscirpenol (DAS), and neosolaniol (NEO), as the main metabolites of FS and FP. We observed statistically significant decreases in the PEF-treated variants for almost all mycotoxin/producer combinations, i.e., for DON in FC isolates and for ZEA in both FC and FG isolates. As concerns the FS and FP strains, a statistically significant reduction could be observed for DAS and NEO (see Figure 5). Considering that the presence of potential degradation products of unknown toxicity could pose a risk to food safety, we also performed targeted screening for PEF-induced transformation/degradation products of mycotoxins according to Stranska et al. [20]. As no convincing signals for these degradation products meeting the standard quality of mass spectrometric data were identified, we can say that the reduced mycotoxin content after PEF treatment is a direct consequence of the reduced viability of the fungal producer.

## 3. Discussion

Rapidly increasing advances and innovations in mass spectrometry, including throughput, sensitivity, resolution, and other parameters, are responsible for generating large amounts of big and very complex data. In response to that, adequate innovations in data processing are required [22]. Currently, it is possible to observe a noticeable trend in the use of freely available “vendor-free” software for data handling, which is continuously driven and upgraded by an active scientific community. Unlike commercial software provided by vendors, open access software is able to support datasets from different instruments and provide readily available data analysis on personal computers [23,24,25]. Data processing software assuring peak picking and alignment (e.g., MS-DIAL [23], MzMine [22]) often works hand in hand with annotating software (e.g., MS-FINDER [26], SIRIUS [27]), providing automatic identification of thousands of compounds. In addition to this, various data “cleaning” software platforms eliminating insignificant features, like ghost peaks, blank ions, and artefacts and ions with improbable retention time shifts or relative mass defects (e.g., MS-CleanR [28], notame [29]), can be used. The outputs of these software platforms then represent a filtered dataset of metabolomic features describing the biological systems examined. Especially thanks to automatic identification, which represents a significant time saving step (instead of manually searching the databases), it is possible to focus on data interpretation and strengthen the impact of the whole study.

In this study, the workflow pipeline, including the MS-DIAL–MS-CleanR–MS-FINDER vendor-free software platform, was used to handle data from processing, through cleaning and filtering, to the final annotation and identification of the features. To describe the metabolomic changes related to PEF treatment and find the biomarkers responsible for these changes, the choice of statistical tools for the selection of statistically significant features was very important. In this way, the use of two independent filters (VIP + ROC) was implemented for the first time. Simultaneous application of these filters provided a list of unique biomarkers related to the up-regulation (PEF-related) and down-regulation (control-related) of various biosynthetic pathways induced by the PEF treatment. In general, this workflow pipeline, based exclusively on free software, enabled the collection of a list of robust and biologically significant metabolites, applicable for studying metabolic changes in a wide range of food-related pathogenic microorganisms.

The primary effect of PEF on a majority of microorganisms is that it creates irreversible micropores on the membranes of microbial cells, rendering the membrane semipermeable, and resulting in cell death. However, different mechanisms probably work in the case of spores, as highly durable forms of microorganisms resistant to a variety of adverse conditions, such as heat or UV/ionizing irradiation, among others. For spores, only a limited inactivation effect has been reported to date, and the possible modes of action are still unclear. There are some studies in the literature focused on bacterial and yeast spores describing changes in their physical shape, and the creation of tiny physical cracks on the spore surface, influencing the two-way transport of nutrients, metabolites, and various signal molecules [30,31]. In our study, the research was focused on the spores of fungi; however, we presume that the PEF-induced processes influencing skeletal changes are more or less similar. The conidia of *Fusarium* fungi are known to contain a high content of endogenous lipids, which usually serve as a good substrate for their germination [32]. However, the PEF-initiated damage to the spore wall probably enables the leakage of these storage lipids (and other nutrients) from spores through the cracks, thus influencing the spore germination, as well as the whole metabolism during fungal maturation and mycelial development. This phenomenon is nicely demonstrated by our data presented in Table S5 where we clearly prove a significantly higher amount of lipid species in the PEF-treated spore suspension when compared to the control (experiment described below Table S5 in the Supplementary Materials).

As further illustrated above and shown in Figure 1 and Figure 2, the metabolomes of the PEF-treated and control *Fusarium* fungi are completely different for all of the *Fusarium* species investigated. By using all of the above-described utilities of contemporary modern science, we were able to identify several very strong and unique PEF-related biomarkers occurring exclusively in the PEF-related samples (see the boxplots in Figure 4), which co-occurred in more than one or two *Fusarium* species. The most frequent PEF-related biomarker occurring in even three of the four *Fusarium* species investigated (FG+FS+FP) was identified as a derivative of glycerolphosphate and propoxy-hydroxy-hexadecanoic acid (Compound^1^ in Table 2 and Figure 4). Despite the deep-seated aim to find any details about this compound, we were not successful in searching the databases, and thus the biological interpretation is not known. Another PEF-related biomarker identified in the FG and FP species was tentatively identified as desotamide D (Table 2, Figure 4), a compound belonging to the cyclic hexapeptide class. The compound was previously isolated from the marine bacterium *Streptomyces scopuliridis* and was shown to be active against some species of pathogenic bacteria [33]. Another PEF-related biomarker co-occurring in the FP and FS species was megalomicin C1 (Table 2, Figure 4), a compound previously described as a secondary metabolite of the micromycete *Micromonospora megalomicea*, and belonging to the macrolide group of antibiotics with a broad scope of antimicrobial activities [34]. Another three so far undescribed compounds belonging to the chemical classes pyranocoumarins, carbazoles, and guanidines (Compound^2^, Compound^3^, Compound^4^; Table 2, Figure 4) were identified as unique PEF-related metabolites occurring in the FG + FS species.

Elucidation of the origination of these biomarkers at the biochemical level is difficult, as the current knowledge of metabolomic pathways for microorganisms, specifically *Fusarium* fungi, is not deep enough (it has been reported that filamentous fungi express tens thousands of secondary metabolites, where perhaps less than 10% of these metabolites are known [35]). Nevertheless, despite the fact that a satisfactory interpretation is not possible at this stage, it is highly probable that these results provide an important basis for follow-up research integrating other superior levels of the “omics” disciplines, including transcriptomics and proteomics. The *Fusarium* metabolites identified in this study were formed exclusively as a consequence of the PEF treatment of the *Fusarium* spores and are directly associated with a significant decrease in organism viability, as well as with a decreased potential of fungi to produce toxic mycotoxins. The current results will serve as cornerstones for further understanding.

## 4. Conclusions

This study aimed to describe the effect of PEF on the changes in the metabolomes of four of the most commonly occurring *Fusarium* species, together with an assessment of the viability and toxigenicity of these micromycetes after the PEF treatment. The most significant results relevant to the practical impact of PEF on the final fungi are summarized below:

- The PEF treatment significantly reduced the viability of the *Fusarium* species investigated in this study (i.e., *F. culmorum*, *F. graminearum*, *F. sporotrichioides*, and *F. poae*), where the extent of the reduction was the highest for *F. poae* (9.4% of surviving spores) and the lowest for *F. culmorum* (53.4% of surviving spores).
- The reduction in *Fusarium* species viability corresponded with a reduction in the mycotoxin content, specifically for DON in the *F. culmorum* isolates, ZEA in both the *F. culmorum* and *F. graminearum* isolates, and DAS and NEO in both the *F. sporotrichioides* and *F. poae* species.
- The overall metabolomes of the PEF-treated and untreated *Fusarium* species were significantly changed, and the statistically significant markers related to the PEF treatment were characterized using the tools of modern high-resolution mass spectrometry. Despite the fact that the biological interpretation of these markers was not successful at this stage, as the knowledge on complex metabolic pathways and the secondary metabolism of micromycetes, including *Fusarium*, is very poor, the well-characterized compounds directly associated with the PEF intervention will serve as a basis for follow-up multi-omics studies.

## 5. Materials and Methods

### 5.1. Analytical Standards and Chemicals

Altogether, 22 certified analytical standards of mycotoxins and their metabolites (Table S6 in the Supplementary Materials) were obtained from Romer labs (Getzersdorf, Austria), Cayman Chemical (Ann Arbor, MI, USA), Merck (Darmstadt, Germany), and LKT Laboratories (St. Paul, MN, USA). This group of analytes included 22 *Fusarium* toxins (for details, see the Supplementary Text).

Acetonitrile, methanol, isopropanol, ammonium formate, ammonium acetate, and formic acid (all LC-MS-grade) were obtained from Merck (Darmstadt, Germany). Deionized water (18 MΩ, 25 °C) was prepared using a Milli-Q^®^ system (Millipore, Bedford, MA, USA).

### 5.2. PEF Treatment of Fusarium Spores

Four highly pathogenic *Fusarium* strains (*F. culmorum*—VURV-F 425; *F. graminearum*—VURV-F 361; *F. poae*—VURV-F 996; *F. sporotrichioides*—VURV-F 205) obtained from the Culture collection of microorganisms of the Crop Research Institute (Czech Republic) were used for the experiments in our study. For each *Fusarium* strain, spore suspensions in sterile tap water (10^5^ spore·mL^−1^) were prepared according to the method described by Šíp et al. [36] and treated separately, one after another, by the PEF System (OMNIPEF, VITAVE, Czech Republic) with a continual treatment chamber reactor. Treatment was performed under the following conditions: voltage 10 kV.cm^−1^, current 10 A, bipolar pulses (positive and negative), pulse width 5 µs, frequency 450 Hz, and flow rate 5 mL·s^−1^. The total treatment time was about 10 min, and the total specific energy delivered to the suspension was 90 J·mL^−1^ (calculated according to Formula S1 in the Supplementary Materials), representing an energy dose similar to real conditions when using PEF to minimize *Fusarium* during food processing (Prusova et al. [37]). The control samples were passed through the PEF device without the application of electric pulses to ensure the same “processing background” for the experiment. At the end of the treatment, the suspension was drained from the device and collected in sterile tubes for further analysis. The device was sterilized prior to each treatment. 

### 5.3. PDA Plate Preparation

Treated and untreated water spore suspensions were subsequently pipetted onto Petri dishes with potato dextrose agar (PDA, HiMedia Laboratories, Brno, Czech Republic). The spore concentration was unified for all samples. Every PDA plate (60 mm) was inoculated with 20 µL of spore suspension and cultivated for seven days at 20 °C in the dark. All sample variants were prepared in eight replicates. To ensure the suppression of bacterial growth, the antibiotic gentamycin (2 mL·L^−1^) was added to the PDA medium. “PDA blank” plates without fungal inoculation were also prepared for the purposes of UHPLC-HRMS/MS data processing. Samples were stored at −80 °C before processing in the laboratory. 

### 5.4. Exraction of PDA Plates

Before extraction, whole frozen samples of PDA plates with mycelial isolates were cut and placed into a PTFE (polytetrafluoroethylene) cuvette to ensure the processing of all fungal mycelia. Then, methanol (MeOH) was added to each sample at a sample/solvent ratio of 1:5 (g:mL) and homogenized (IKA-Turrax-T25D, IKA Works GmbH & Co. KG, Staufen im Breisgau, Germany). The cuvette with the homogenized sample was then placed on a laboratory shaker (HS 260 basic, IKA Works GmbH & Co. KG, Germany) for 30 min (shaking at 240 RPM). The extract was then centrifuged (Rotina 35 R, Hettich Zentrifugen, Germany) for 2 min at 13,528 RCF (relative centrifugal force) and microfiltered through 0.2 µm spin filters (Ciro, Deerfield Beach, FL, USA). Finally, an aliquot of approx. 1 mL was transferred to a glass HPLC vial for further analyses by UHPLC-HRMS/MS. The “PDA blank” samples were prepared in the same way. To eliminate potential system drift in the analytical system, the pooled extract (quality control sample (QC)) was prepared by mixing 100 µL of extract from each sample and analyzed. To exclude background signals from the laboratory dishes and solvents, a “processing blank” sample was prepared together with the analyzed samples.

### 5.5. UHPLC-HRMS/MS Metabolomic Fingeprinting

UHPLC-HRMS/MS analysis was performed according to the study of Stranska et al. [38], with slight modifications. For more details about the UHPLC-HRMS/MS method, see the Supplementary Text. To record the MS1 and MS/MS data, the TOF MS method (full scan) and the Information Dependent Acquisition (IDA) method with electrospray ionization in positive and negative polarity (ESI+/ESI-) were used. The working range was 100–1200 *m*/*z* for MS1 and 50–1000 *m*/*z* for MS/MS, and data were acquired between 0.5 and 19 min. The MS/MS spectra were collected for the eight most intensive ions of the MS spectra throughout the chromatographic run. The collision energy was 35 ± 15 V, and the QC sample was analyzed every ten samples during the sequence. Instrument control and data acquisition were operated with Analyst 1.7.1 TF software (SCIEX, Concord, ON, Canada), and qualitative analysis was performed using SCIEX OS software (v. 1.5.0.23389, SCIEX, Concord, ON, Canada).

### 5.6. Metabolomic Data Processing and Statistical Analysis

*Data processing*: For data mining and processing, the freely available MS-DIAL–MS-CleanR–MS-FINDER software platform was used according to the workflow published by Stranska et al. [38]. In the first step, the UHPLC-HRMS/MS data were processed and deconvoluted by MS-DIAL (v. 4.80, 2021, RIKEN, Saitama, Japan). For peak picking, the MS1 and MS/MS tolerances were set to 0.03 and 0.1 Da in the centroid mode for both the datasets acquired in the ESI- and ESI+ modes. For peak alignment, the QC reference file was used, with an RT tolerance of 0.05 min and mass tolerance of 0.015 Da. The minimum peak height for peak detection was set to amplitude 9000 (referring to 70% under the observed baseline for a blank injection). Zero values were replaced with 1/10 of the minimum peak height for all samples during the export of the aligned results from MS-DIAL. In the second step, the aligned results were cleaned with the MS-CleanR (MetaToul-AgromiX Platform) tool based on the user-friendly Shiny–RStudio interface. For data cleaning, all filters, including blank filters, a ghost peak filter, incorrect mass, relative standard deviation, and relative mass defect, were activated, with the minimum blank ratio set to 0.8, the maximum relative standard deviation (RSD) set to 30%, and the relative mass defect (RMD) ranging from 50 to 3000. The maximum RT difference and mass difference tolerance values were set to 0.025 min and 0.005 Da for Pearson correlation and ESI+/- data merging. In this step, feature clustering based on Pearson correlation and the MS-DIAL peak character estimation algorithm (MS-DIAL-PCE) was implemented, and one peak (the most intense) was kept in each cluster. In the third step, the filtered features were automatically annotated with MS-FINDER (v. 3.52, 2021, RIKEN, Japan). The MS1 and MS/MS tolerances were set to 5 and 15 ppm, respectively. C, H, O, N, P, and S atoms were included in the formula finder parameter module. As a data source for automatic identification, the databases YMDB, ECMDB, PlantCyc, ChEBI, T3DB, NPA, KNApSAcK, LipidMaps, and PubChem were used. On the basis of the accurate mass and isotope ratio of MS1 ions, the elemental compositions of the candidate ions were proposed, and possible chemical structures of the candidates were computed by comparing the experimental and in silico MS/MS spectra (MS-FINDER in silico fragmentation is based on the hydrogen rearrangement rules published by Tsugawa et al. [26]). For the last step, MS-FINDER offers several available chemical formulas and related chemical structures ranked by score for each parameter. The best results often match with the highest scoring formula and structure options and are included in the next steps. Finally, MS-CleanR was launched, where it matched information from the MS-FINDER automatic annotation with filtered features and created a final data matrix with filtered and annotated features for further statistical analysis. In this step, MS-CleanR also matched data from the ESI+ and ESI- ionization modes into one data matrix. First, the data were processed together in order to obtain a primary insight, and then separately for each *Fusarium* species (FC, FG, FP, FS). *Statistical analysis*: Each data matrix (FC, FG, FP, FS) was filtered using univariate tools (volcano plot—*t*-test, fold change) to exclude statistically insignificant features and prevent overfitting of the final models. Univariate methods were performed by using the freely available web platform MetaboAnalyst (v. 5.0, Xia Lab, McGill, Montreal, QC, Canada), with a *t*-test false discovery rate (FDR) adjusted *p*-value of <0.01 and fold change of >2. A general overview of the features reduction during data filtration for all data matrices is summarized in Table S7. The final filtered and annotated data matrices, including annotated features only (for each species separately), were processed with MS Excel to perform normalization of the data (total area sum normalization). Normalized data were uploaded to SIMCA software (v. 17.0, 2021, Sartorius, Göttingen, Germany), where multivariate principal component analysis (PCA) and orthogonal partial least squares discriminant analysis (OPLS-DA) were performed. Before the creation of every classification model, Pareto scaling and logarithmic transformation of the data were performed. The quality of the models was determined using the R^2^Y and Q^2^ validation parameters calculated by a 7-round internal cross-validation and misclassification table (MT), which summarizes how well the selected models classify the samples into the known classes. Candidate biomarker compounds were selected based on OPLS-DA S-plots and Variable Importance in Projection (VIP) plots, together with receiver operating characteristics (ROCs). Both filters were applied simultaneously in an orthogonal way to assure proper statistical filtration and to keep only the strong biomarkers relevant for further data interpretation. For selection, VIP scores greater than 1 (VIP score >1) and ROC area under the curve (AUC = 1) parameters were set. The biomarker selection process was confirmed by checking the boxplots illustrating the distribution of features between the sample groups.

### 5.7. Target Analysis of Mycotoxins by UHPLC-HRMS/MS and Statistical Evaluation

For targeted screening of mycotoxins, methanolic extracts of PDA plates (Section 5.4, Extraction of PDA Plates) were used. UHPLC-HRMS/MS analysis was performed according to the study of Dzuman et al. [39]. The UHPLC-HRMS/MS method is described in detail in the Supplementary Text and in Tables S8 and S9. For the semi-quantitative results, an external solvent (methanol) calibration series of mycotoxin standards (Section 5.1, Analytical Standards and Chemicals) was created in the concentration range of 0.5–200 ng·mL^−1^. The determined limits of quantification (LOQs) of all detected mycotoxins are summarized in Table S10. The significance of the differences between the PEF-treated and control samples was statistically evaluated using the Wilcoxon rank-sum test (*p*-value < 0.1) performed in MetaboAnalyst.

### 5.8. Viability Test and Statistical Evaluation

*Fusarium* spore viability testing was performed on PDA plates prepared according to Section 5.3, PDA Plate Preparation. The number of colony-forming units (CFUs) per plate was counted after 3 days of cultivation. Statistical differences between the PEF-treated and control samples were analyzed by ANOVA (analysis of variance) with a 95% confidence interval and 5% level of significance separately for each *Fusarium* species. ANOVA was performed using STATISTICA software (ver. 14.0.0.15, TIBCO, Palo Alto, CA, USA).

## Figures and Tables

**Table 1:** Number of metabolomic features according to their polarity gained from UHPLC-HRMS/MS records of various extraction solvents and their mixtures. Electrospray ionization in positive (ESI+) and negative (ESI-) polarity.

Polarity of Features	Extraction Solvent/Solvent Mixtures	Number of Features		
		ESI+	ESI-	SUM.
Polar (0–6 min Rt)	water	4179	2962	7141
	methanol/water (50:50, *v*/*v*)	5822	4165	9987
	methanol	6208	4179	10,387
	methanol/propan-2-ol (50:50, *v*/*v*)	5790	3798	9588
Middle-polar (6–12 min Rt)	water	1517	197	1714
	methanol/water (50:50, *v*/*v*)	1191	178	1369
	methanol	4466	1285	5751
	methanol/propan-2-ol (50:50, *v*/*v*)	4738	1272	6010
Nonpolar (12–19 min Rt)	water	1416	15	1431
	methanol/water (50:50, *v*/*v*)	899	9	908
	methanol	1435	10	1445
	methanol/propan-2-ol (50:50, *v*/*v*)	2480	149	2629

**Table 2:** PEF-related biomarkers co-occurring in at least two *Fusarium* species (sorted according to overlays between each dataset: FC, FG, FP, FS).

Dataset Overlays	MS/MS spec. Match Score *	Trend	Structure	Ontology	InChIKey Chemical Structure
FG ∩ FP ∩ FS	1	PEF	Compound ^1^	Oxidized long-chain fatty acid derivatives	IGLRXLRPYYHFQN-UHFFFAOYSA-N 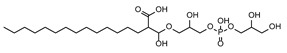
FG ∩ FP	3	PEF	Desotamide D	Cyclic hexapeptides	LZYIIJVZWQGRJH-CBHSLMNUSA-N 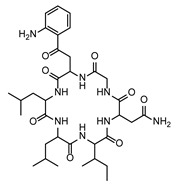
FP ∩ FS	1	PEF	Megalomicin C1	Macrolides	NGOSGEYHKQYUTN-XIBKBKGSSA-N 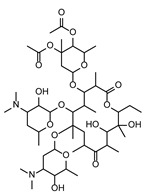
FG ∩ FS	1	PEF	Compound ^2^	Angular pyranocoumarins	PRZGTCWIDNLJEN-UHFFFAOYSA-N 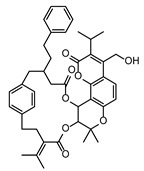
FG ∩ FS	2	PEF	Compound ^3^	Carbazoles	CBMRXUBLKLUUQW-CHRKOGDLNA-N 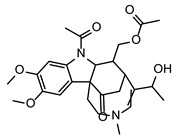
FG ∩ FS	3	PEF	Compound ^4^	Guanidines	YMMKPCXKLMUKEC-UHFFFAOYSA-N 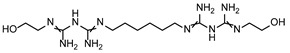

* MS/MS spectrum match score: “0” = no fragments, “1” = only one fragment referring to pseudomolecular ion, “2” = one fragment, “3” = two or more fragments. ^1^ 2-((3-(((2,3-dihydroxypropoxy)(hydroxy)phosphoryl)oxy)-2-hydroxypropoxy)(hydroxy)methyl)hexadecanoic acid. ^2^ 13-(hydroxymethyl)-19,19-dimethyl-3-(2-phenylethyl)-12-(propan-2-yl)-23-(propan-2-ylidene)-6,10,18,21-tetraoxapentacyclo [24.2.2.0^7^,^20^.0^8^,^17^.0^9^,^14^]triaconta-1(28),8,12,14,16,26,29-heptaene-5,11,22-trione. ^3^ [8-acetyl-12-(1-hydroxyethyl)-4,5-dimethoxy-14-methyl-17-oxo-8,14-diazatetracyclo[9.5.2.0¹,⁹.0²,⁷]octadeca-2(7),3,5,12-tetraen-10-yl]methyl acetate. ^4^ 1-[N′-[6-[[amino-[[N′-(2-hydroxyethyl)amidino]amino]methylene]amino]hexyl]amidino]-2-(2-hydroxyethyl)guanidine.

## Data Availability

The original contributions presented in this study are included in the article/supplementary material. Further inquiries can be directed to the corresponding author.

## Supplementary Materials

The following supporting information can be downloaded at https://www.mdpi.com/article/10.3390/toxins17010033/s1: Supplementary text to Section 5.1, Analytical Standards and Chemicals. Supplementary text to Section 5.2, PEF Treatment of *Fusarium* Spores. Supplementary text to Section 5.5, UHPLC-HRMS/MS Metabolomic Fingerprinting. Supplementary text to Section 5.8, Target Screening of Mycotoxins by UHPLC-HRMS/MS. Table S1: Quality parameters (R2Y, Q2) and misclassification table (MT) results of OPLS-DA models. Table S2: Control-related biomarkers and their ontologies co-occurring in at least two *Fusarium* species. Table S3: Boxplots demonstrating statistically significant differences between control (C) and PEF-treated spore suspensions of four *Fusarium* species (FC—*F. culmorum*; FG—*F. graminearum*; FP—*F. poae*; FS—*F. sporotrichioides*), CFU/dish (colony-forming units per Petri dish with PDA). Significance was tested using ANOVA (*p*-value <0.5). Table S4: List of semi-quantified mycotoxins with their abundance in selected samples (3 biological replicates of each *Fusarium* species (FC—*F. culmorum*; FG—*F. graminearum*; FP—*F. poae*; FS—*F. sporotrichioides*) and sample type: control vs. PEF-treated). Results represent concentrations of mycotoxins (15-acetyldeoxynivalenol (15-ADON), 3-acetyldeoxynivalenol (3-ADON), beauvericin (BEA), diacetoxyscirpenol (DAS), deoxynivalenol (DON), HT-2 toxin (HT2), neosolaniol (NEO), T-2 toxin (T2), zearalenone (ZEA)) [µg·kg^−1^] spread in the whole PDA plate, including the PDA media. Mycotoxins with statistically significant differences (Wilcoxon rank-sum test (*p*-value < 0.1)) are highlighted green. Table S5: Non-targeted lipidomics by UHPLC-HRMS/MS—number of nonpolar features representing amount of lipid species in control and PEF-treated spore suspensions. Table S6: List of 22 certified analytical standards of mycotoxins and their metabolites. Table S7: The general overview of the feature reduction during data filtration for all data matrices. Table S8: Detailed conditions of UHPLC-HRMS/MS mycotoxin analysis. Table S9: A list of exact masses of target analytes, fragment ions, retention times, and analyte-specific normalized collision energies (NCEs). Precursor ions for fragmentation are in bold. Table S10: Determined limits of quantification (LOQs) of all semi-quantified mycotoxins in PDA plates. Figures S1–S4: UHPLC-HRMS fingerprints of PEF-treated (blue) and control (green) *F. culmorum* (FC), *F. graminearum* (FG), *F. poae* (FP), and *F. sporotrichioides* (FS) samples; MeOH extracts, ESI+. The zoomed-in areas of the TIC chromatograms highlight the differences in the low-intensity regions. Figure S5: The OPLS-DA models (score scatter plot) of each *Fusarium* species dataset (FC—*F. culmorum*; FG—*F. graminearum*; FP—*F. poae*; FS—*F. sporotrichioides*) colored according to PEF treatment (PEF) and control with excellent values of quality parameters.
