# Supplementary material for: Pulsed Electric Field Induces Significant Changes in the Metabolome of Fusarium Species and Decreases Their Viability and Toxigenicity
Source: Toxins (Basel). 2025 Jan 11;17(1):33. doi: 10.3390/toxins17010033 (PMC11769547; doi:10.3390/toxins17010033)
Supplement: Supplementary file 1 [file toxins-17-00033-s001.zip › toxins-3366338-supplementary.pdf]

Supplementary Materials: Pulsed Electric Field Induces Significant Changes in the Metabolome of *Fusarium* Species and Decreases Their Viability and Toxigenicity

Adam Behner, Jana Palicova, Anna-Hirt Tobolkova, Nela Prusova and Milena Stranska

Supplementary text to Section 5.1 Analytical Standards and Chemicals

22 certified analytical standards of mycotoxins and their metabolites

The group of 22 *Fusarium* toxins specifically included: 15-acetyldeoxynivalenol (15-ADON), 3-acetyldeoxynivalenol (3-ADON), beauvericin (BEA), deoxynivalenol (DON), deoxynivalenol-3-glucoside (D3G), diacetoxyscirpenol (DAS), enniatins A, A1, B, B1 (Enn-A, Enn-A1, Enn-B, Enn-B1), fumonisins B1, B2, B3 (FB1, FB2, FB3), fusarenon x (FUS-X), HT-2 toxin (HT2), neosolaniol (NEO), nivalenol (NIV), T-2 toxin (T2), verruculogen (VER), zearalenone (ZEA) α- and β-zearalenol (α-ZOL, β-ZOL). The range of declared purity of all analytical standards was between 95.30% and 100.00%. For the purpose of calibration experiments, a working mixture of all standards was freshly prepared in acetonitrile at concentration 1000 $\mu g.L^{-1}$.

Supplementary text to Section 5.2 PEF Treatment of *Fusarium* Spores

The total specific energy calculation delivered to the suspension of spores

$$Q = \frac{U \times I \times t \times f}{q} \qquad \textbf{(Formula S1)}$$

Formula S1 was used to calculate the specific energy delivered in $J.mL^{-1}$ each PEF treatment, where Q = the specific energy delivered ($J.mL^{-1}$); U = the voltage set on the screen (V); I = the measured current (A); t = the width of 1 pulse (s); f = set frequency (Hz) (bipolar pulses are taken into account by multiplying frequency by two) and q = volume flow rate of sample ($mL.s^{-1}$) (flow rate of treated suspension 5 $mL.s^{-1}$).

Supplementary text to Section 5.5 UHPLC-HRMS/MS Metabolomic Fingerprinting

UHPLC-HRMS/MS method

For metabolite separation, the UHPLC system (Dionex UltiMate 3000 RS UHPLC system; Thermo Fisher Scientific, Waltham, USA) equipped with an Acquity UPLC® BEHC18 reverse phase column (100 mm x 2.1 mm; 1.7 µm; Waters, MA, USA) was used. The injection volume was 2 µl, autosampler temperature was 10°C and the column temperature was 60°C. The mobile phase consisted (A) 5 mM ammonium formate in a mixture of Milli-Q water:methanol (95:5, *v/v*) with 0.1% formic acid (*v/v*) and (B) 5 mM ammonium formate in a mixture of isopropanol:methanol:Milli-Q water (65:30:5, *v/v/v*) with 0.1% formic acid (*v/v*). In both negative and positive ionization modes, the following elution gradient was used: 0.0 min (10% B; 0.4 $mL.min^{-1}$), 1.0 min (50% B; 0.4 $mL.min^{-1}$), 5.0 min (80% B; 0.4 $mL.min^{-1}$), 11.0 min (100% B; 0.4 $mL.min^{-1}$), 19.0 min (100% B; 0.4 $mL.min^{-1}$), 19.1 min (10% B; 0.4 $mL.min^{-1}$), 21.0 min (10% B; 0.4 $mL.min^{-1}$). For detection, the SCIEX TripleTOF® 6600 quadrupole time-of-flight mass spectrometer (SCIEX, Concord, ON, Canada) was used in both negative (ESI-) and positive (ESI+) ionization modes. In the ESI- mode, the parameters of the ion source were: curtain gas pressure: 35 psi; nebulizing gas pressure: 55 psi; drying gas pressure: 55 psi; temperature: 500°C; capillary voltage: -4.5 kV; and declustering potential: 80 V. The capillary voltage in ESI+ was +4.5 kV; other parameters were the same as for ESI-. The calibration delivery system (CDS) allowed an automatic *m/z* calibration of the MS system every 10 samples in sequence using

a negative or positive APCI calibration solution (SCIEX, Concord, ON, Canada). The resolving power was >40,000 FWHM, *m/z* 829.5393 for ESI+ and *m/z* 933.6370 for ESI-.

Supplementary text to Section 5.8 Target Screening of Mycotoxins by UHPLC-HRMS/MS

The UHPLC-HRMS/MS method in detail

For mycotoxin separation, the UHPLC system (Dionex UltiMate 3000 RS UHPLC system; Thermo Fisher Scientific, Walthman, USA). For the detection, the Q-Exactive Plus™ (Thermo Scientific, USA) quadrupole-orbitrap high-resolution tandem mass spectrometer was used in both ESI+ and ESI- ionization modes. Detailed conditions of UHPLC-HRMS/MS analysis are summarized in **Table S8**. Acquisition of both ESI+ and ESI- data was performed in full-spectral mode with conditional fragmentation of a total of 22 mycotoxins for confirmatory purposes, i.e. in the full MS-data dependent MS2 (fullMS-ddMS2) acquisition mode. A list of exact masses of target analytes, fragment ions, retention times and analyte specific normalized collision energies (NCE) is available in **Table S9**.

Table S1. Quality parameters (R^2Y, Q^2) and misclassification table (MT) results of OPLS-DA models.

Dataset	R^2Y	Q^2	Correct percentage (MT) PEF/control
FC	0.998	0.981	100% / 100%
FG	0.985	0.959	100% / 100%
FP	0.994	0.951	100% / 100%
FS	0.973	0.928	100% / 100%

Table S2. Control-related biomarkers and their ontologies co-occurring in at least two *Fusarium* species.

Dataset	MS/MS spec. match score*	Trend	Structure	Ontology	InChIKey
FS	1	control	Sarsasapogenin benzoate	Triterpenoids	GEHXUPXZDCGVMC-UHFFFAOYSA-N
FS	2	control	Compound[1]	Triterpenoids	WKCHWHAYABLGGH-NOEDMIOANA-N
FC	1	control	Gypsophilin	Triterpenoids	YJZUFDBGHBUWHO-MHGBACFYNA-N
FC	3	control	Spumigin H	N-acyl-alpha amino acids and derivatives	VBJAVXUALMEUNC-SLRGTUBMSA-N
FC	1	control	Spumigin G	N-acyl-alpha amino acids and derivatives	YGAGCUYKOBGAQX-SYCDWYGJSA-N
FS	1	control	UCF 116C	N-acyl-alpha amino acids and derivatives	XCJPPAFCCOSJBT-QAIGDSDZNA-N
FC	3	control	Apetaline A	Oligopeptides	FADRDHDLIJSOGO-AKCUSSKQNA-N
FG	2	control	Gassericin B2	Oligopeptides	AVEZLZYNAVAHHU-UMJHPYLWSA-N

*MS/MS spectrum match score: 0 = no fragments, 1 = only one fragment referring to pseudomolecular ion, 2 = one fragment, 3 = two or more fragments.

[1]N-{15-[1-(dimethylamino)ethyl]-14-hydroxy-7-(hydroxymethyl)-7,12,16-trimethyl-18-oxopentacyclo[9.7.0.0^{1},^{3}.0^{3},^{8}.0^{12},^{16}]octadecan-6-yl}benzamide

Table S3. Boxplots demonstrating statistically significant differences between control (C) and PEF-treated spore suspensions of four *Fusarium* species (FC—F. *culmorum*; FG—F. *graminearum*; FP—F. *poae*; FS—F. *sporotrichioides*), CFU/dish (colony-forming units per Petri dish with PDA). Significance was tested using ANOVA (p-value <0.5).

Dataset (*Fusarium* species)	**FC**	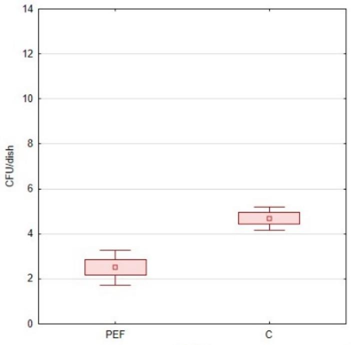
	**FG**	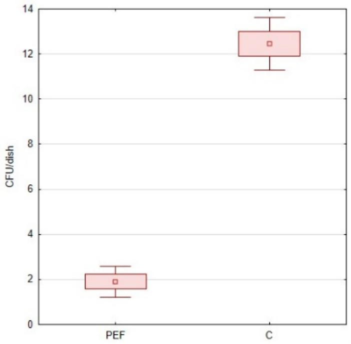
	**FP**	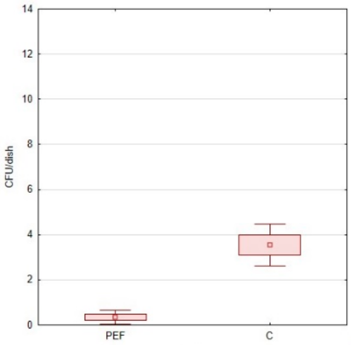
	**FS**	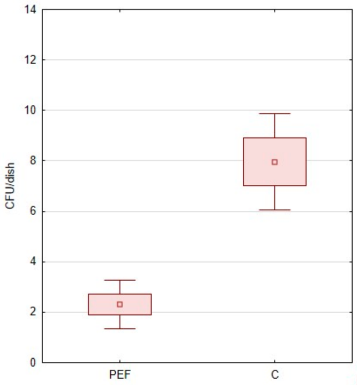

Table S4: List of semi-quantified mycotoxins with their abundance in selected samples (3 biological replicates of each *Fusarium* species (**FC** – *F. culmorum*, **FG** – *F. graminearum*, **FP** – *F. poae*, **FS** – *F. sporotrichioides*) and sample type: control vs. PEF-treated). Results represent concentrations of mycotoxins (15-acetyldeoxynivalenol (**15-ADON**), 3-acetyldeoxynivalenol (**3-ADON**), beauvericin (**BEA**), diacetoxyscirpenol (**DAS**), deoxynivalenol (**DON**), HT-2 toxin (**HT2**), neosolaniol (**NEO**), T-2 toxin (**T2**), zearalenone (**ZEA**)) [μg.kg^{-1}] spread in whole PDA plate including PDA media. Mycotoxins with statistically significant differences (Wilcoxon rank-sum test (p-value <0.1)) are highlighted green.

	Concentration of mycotoxin [μg.kg^{-1}]								
Sample	**15-ADON**	**3-ADON**	**BEA**	**DAS**	**DON**	**HT-2**	**NEO**	**T-2**	**ZEA**
**FC_control_01**	1333	1711	–	19	51	–	–	–	6
**FC_control_02**	881	1154	–	15	39	–	–	–	3
**FC_control_03**	995	1303	–	19	46	–	–	–	4
**FC_PEF_01**	1156	1556	–	19	35	–	–	–	1
**FC_PEF_02**	986	1194	–	14	27	–	–	–	1
**FC_PEF_03**	<LOQ	<LOQ	–	<LOQ	<LOQ	–	–	–	<LOQ
**FG_control_01**	–	–	–	–	–	–	–	–	54
**FG_control_02**	–	–	–	–	–	–	–	–	50
**FG_control_03**	–	–	–	–	–	–	–	–	33
**FG_PEF_01**	–	–	–	–	–	–	–	–	28
**FG_PEF_02**	–	–	–	–	–	–	–	–	27
**FG_PEF_03**	–	–	–	–	–	–	–	–	29
**FP_control_01**	–	–	74	287	–	–	7	–	–
**FP_control_02**	–	–	43	733	–	–	18	–	–
**FP_control_03**	–	–	33	546	–	–	10	–	–
**FP_PEF_01**	–	–	132	34	–	–	1	–	–
**FP_PEF_02**	–	–	8	112	–	–	2	–	–
**FP_PEF_03**	–	–	10	30	–	–	1	–	–
**FS_control_01**	–	–	3	233	–	52	594	8332	–
**FS_control_02**	–	–	5	201	–	32	427	7173	–
**FS_control_03**	–	–	3	244	–	58	615	9743	–
**FS_PEF_01**	–	–	7	145	–	41	306	5690	–
**FS_PEF_02**	–	–	1	200	–	33	396	7473	–
**FS_PEF_03**	–	–	3	195	–	38	387	6945	–

Table S5: Non-targeted lipidomics by UHPLC-HRMS/MS – number of nonpolar features representing amount of lipid species in control and PEF-treated spore suspensions; experimental details provided below *

Spore suspension	Number of lipids (12-19 min Rt), ESI+/-
Control (n=3)	211±21
PEF-treated (n=3)	334±46

* *Aqueous Fusarium spore suspension (mixture of all FC, FG, FS and FP) was prepared and treated as described in section* ***5.2 PEF treatment of Fusarium spores****. After the treatment, 50 mL of the suspension was extracted into 1 mL of isooctane (3 hours shaking on an automatic shaker 240 rpm). After phases separation, 200 µL of isooctane was transferred into LC-MS vial, evaporated with a gentle stream of nitrogen, redissolved in the same volume of methanol (200 µL), and analyzed by the UHPLC-HRMS/MS method described in section* ***5.5 The UHPLC-HRMS/MS metabolomic fingerprinting****. To exclude background signals from laboratory dishes and solvents, a "processing blank" sample was prepared together with the analyzed samples. Raw LC-MS data were processed according to section* ***5.6 Metabolomic data processing and statistical analysis****. For lipidomic screening, only nonpolar features eluting between 12-19 min retention time (Rt) were included.*

Table S6: List of 22 certified analytical standards of mycotoxins and their metabolites.

No	Mycotoxin	CAS	Producer
**1**	15-Acetyldeoxynivalenol	88337-96-6	Romer Labs
**2**	3-Acetyldeoxynivalenol	50722-38-8	Sigma-Aldrich
**3**	Beauvericin	26048-05-5	Cayman Chemical
**4**	Deoxynivalenol	51481-10-8	Sigma-Aldrich
**5**	Deoxynivalenol-3-glucoside	131180-21-7	Romer Labs
**6**	Diacetoxyscirpenol	2270-40-8	Romer Labs
**7**	Enniatin A	2503-13-1	Sigma-Aldrich
**8**	Enniatin A1	4530-21-6	Cayman Chemical
**9**	Enniatin B	917-13-5	Merck
**10**	Enniatin B1	19914-20-6	Merck
**11**	Fumonisin B1	116355-83-0	Cayman Chemical
**12**	Fumonisin B2	116355-84-1	Cayman Chemical
**13**	Fumonisin B3	136379-59-4	LKT Laboratories
**14**	Fusarenon X	23255-69-8	Romer Labs
**15**	HT-2 toxin	26934-87-2	Merck
**16**	Neosolaniol	36519-25-2	Romer Labs
**17**	Nivalenol	23282-20-4	Romer Labs
**18**	T-2 toxin	21259-20-1	Sigma-Aldrich
**19**	Verruculogen	12771-72-1	Cayman Chemical
**20**	Zearalenone	17924-92-4	Cayman Chemical
**21**	α-zearalenol	364-55-72-8	Cayman Chemical
**22**	β-zearalenol	71030-11-0	Cayman Chemical

Table S7: The general overview of the feature reduction during data filtration for all data matrices.

	Number of features			
Data treatment step	Processing	Filtration	Automatic identification	Statistical filter
Software tool	MS-DIAL	MS-CleanR	MS-FINDER	Metaboanalyst: Volcano plot (FC>2, p-value <0,01, FDR)
FC (ESI+/-)	9465	1476	514	97
FG (ESI+/-)	10,637	1643	571	95
FP (ESI+/-)	9621	1650	571	110
FS (ESI+/-)	9015	1380	480	214

Table S8: Detailed conditions of UHPLC-HRMS/MS mycotoxin analysis.

**Chromatographic separation parameters**	
Name of the system	Dionex UltiMate 3000 RS UHPLC
Column	Acquity® UPLC HSS T3
Injection volume	2 µl
Autosampler temperature	10°C
Column temperature	40°C
Mobile phases	ESI+: 5mM ammonium format and 0.2% formic acid (*v*/*v*) in Milli-Q water (A) and methanol (B)<br>ESI-: 5mM ammonium acetate in Milli-Q water (C) and methanol (D)
Elution gradient	ESI+: 0.0 min (10% B; 0.3 $mL.min^{-1}$), 1.0 min (50% B; 0.3 $mL.min^{-1}$), 8.0 min (100% B; 0.4 $mL.min^{-1}$), 10 min (10% B; 0.3 $mL.min^{-1}$)<br>ESI-: 0.0 min (10% D; 0.3 $mL.min^{-1}$), 1.0 min (50% D; 0.3 $mL.min^{-1}$), 4.0 min (100% D; 0.4 $mL.min^{-1}$), 6.0 min (10% D; 0.3 $mL.min^{-1}$)
**Mass spectrometry detection paramters**	
Name of the system	Q-Exactive Plus™
Sheath/auxiliary gas flow rate	45/10 arbitary units
Capillary temperature	320°C
Heather temperature	300°C
Electrospray voltage	±3.5 kV
S-lens value	55
fullMS parameters	mass range of 100–1200 *m*/*z*, resolving power 70,000 full width at half maximum (FWHM), automatic gain control target (AGC target) $3e^6$, maximum inject time (maxIT) 100 ms
ddMS^2 parameters	mass range from *m*/*z* 50 to *m*/*z* of fragmented analyte (+*m*/*z* 10), resolving power 17,500 FWHM, AGC target $1e^5$, maxIT 50 ms

Table S9: A list of exact masses of target analytes, fragment ions, retention times and analyte-specific normalized collision energies (NCEs). Precursor ions for fragmentation are in bold.

No	Mycotoxin	Summary formula	ESI(–)			ESI(+)			NCE (%)	Fragment 1		Fragment 2	
			$[M-H]^-$	$[M+CH_3COO]^-$	RT (min)	$[M+H]^+$	$[M+NH_4]^+$	RT (min)		Summary formula	m/z	Summary formula	m/z
1	15-Acetyldeoxynivalenol	C17H22O7	337.1293	397.1504	2.77	339.1438	**356.1704**	2.75	10	[C17H23O7]$^+$	339.1438	[C17H21O6]$^+$	321.1333
2	3-Acetyldeoxynivalenol	C17H22O7	337.1293	**397.1504**	2.61	339.1438	356.1704	-	10	[C2H3O2]$^-$	59.0138	[C17H21O7]$^-$	337.1292
3	Beauvericin	C45H57N3O9	782.4022	842.4233	8.06	784.4168	**801.4433**	8.02	30	[C15H18O2N]$^+$	244.1332	[C9H12N]$^+$	134.0964
4	Deoxynivalenol	C15H20O6	295.1187	**355.1398**	2.09	297.1333	314.1598	2.13	10	[C2H3O2]$^-$	59.0139	[C15H19O6]$^-$	295.1187
5	Deoxynivalenol-3-glucoside	C21H30O11	457.1715	**517.1927**	2.01	459.1861	476.2126	2.03	20	[C20H27O10]$^-$	427.1610	[C21H29O11]$^-$	457.1715
6	Diacetoxyscirpenol	C19H26O7	365.1606	425.1817	3.61	367.1751	**384.2017**	3.58	20	[C17H23O5]$^+$	307.1540	[C15H17O2]$^+$	229.1223
7	Enniatin A	C36H63N3O9	680.4492	740.4703	8.44	682.4637	**699.4903**	8.40	20	[C12H20O2N]$^+$	210.1489	[C36H64O9N3]$^+$	682.4637
8	Enniatin A1	C35H61N3O9	666.4335	726.4546	8.28	668.4481	**685.4746**	8.24	20	[C35H62O9N3]$^+$	668.4481	[C12H20O2N]$^+$	210.1489
9	Enniatin B	C33H57N3O9	638.4022	698.4233	7.92	640.4168	**657.4433**	7.88	30	[C11H18O2N]$^+$	196.1332	[C11H20O3N]$^+$	214.1438
10	Enniatin B1	C34H59N3O9	652.4179	712.4390	8.11	654.4324	**671.4590**	8.07	30	[C34H60O9N3]$^+$	654.4324	[C11H18O2N]$^+$	196.1332
11	Fumonisin B1	C34H59NO15	720.3812	780.4023	4.49	**722.3957**	739.4223	4.44	30	[C22H40ON]$^+$	334.3104	[C22H44O3N]$^+$	370.3316
12	Fumonisin B2	C34H59NO14	704.3863	764.4074	5.59	**706.4008**	723.4274	5.53	30	[C22H42ON]$^+$	336.3261	[C22H40N]$^+$	318.3155
13	Fumonisin B3	C34H59NO14	704.3863	764.4074	5.12	**706.4008**	723.4274	5.07	30	[C22H42ON]$^+$	336.3261	[C22H44O2N]$^+$	354.3367
14	Fusarenon X	C17H22O8	353.1242	**413.1453**	2.27	355.1387	372.1653	2.31	10	[C2H3O2]$^-$	59.0139	[C17H21O8]$^-$	353.1242
15	HT-2 toxin	C22H32O8	423.2024	483.2236	4.37	425.2170	**442.2435**	4.33	10	[C15H19O4]$^+$	263.1278	[C14H15O2]$^+$	215.1067
16	Neosolaniol	C19H26O8	381.1555	441.1766	2.37	383.1700	**400.1966**	2.34	10	[C17H21O5]$^+$	305.1384	[C15H17O3]$^+$	245.1172
17	Nivalenol	C15H20O7	311.1136	**371.1348**	1.84	313.1282	330.1547	1.87	10	[C2H3O2]$^-$	59.0139	[C14H17O6]$^-$	281.1031
18	T-2 toxin	C24H34O9	465.2130	525.2341	4.62	467.2276	**484.2541**	4.95	10	[C21H31O7]$^+$	395.2064	[C12H17O4]$^+$	225.1121
19	Verrucarol	C15H22O4	265.1445	325.1657	2.61	**267.1591**	284.1856	2.58	10	[C15H21O3]$^+$	249.1485	[C15H19O2]$^+$	231.1380
20	Zearalenone	C18H22O5	317.1394	377.1606	3.90	**319.1540**	336.1805	5.43	50	[C9H7O]$^-$	131.0502	[C10H7O3]$^-$	175.0401
21	α-zearalenol	C18H24O5	**319.1551**	379.1762	3.79	321.1697	338.1962	5.23	60	[C9H4O3]$^-$	160.0166	[C9H6O]$^-$	130.0424
22	β-zearalenol	C18H24O5	**319.1551**	379.1762	3.57	321.1697	338.1962	4.70	60	[C9H6O]$^-$	130.0424	[C9H4O3]$^-$	160.0166

Table S10: Determined limits of quantification (LOQs) of all semi-quantified mycotoxins in PDA plates.

No	Mycotoxin	LOQ ($ng.mL^{-1}$)
1	15-Acetyldeoxynivalenol	5
2	3-Acetyldeoxynivalenol	0.5
3	Beauvericin	0.5
4	Deoxynivalenol	5
5	Diacetoxyscirpenol	0.5
6	HT-2 toxin	5
7	Neosolaniol	0.5
8	T-2 toxin	1
9	Zearalenone	1

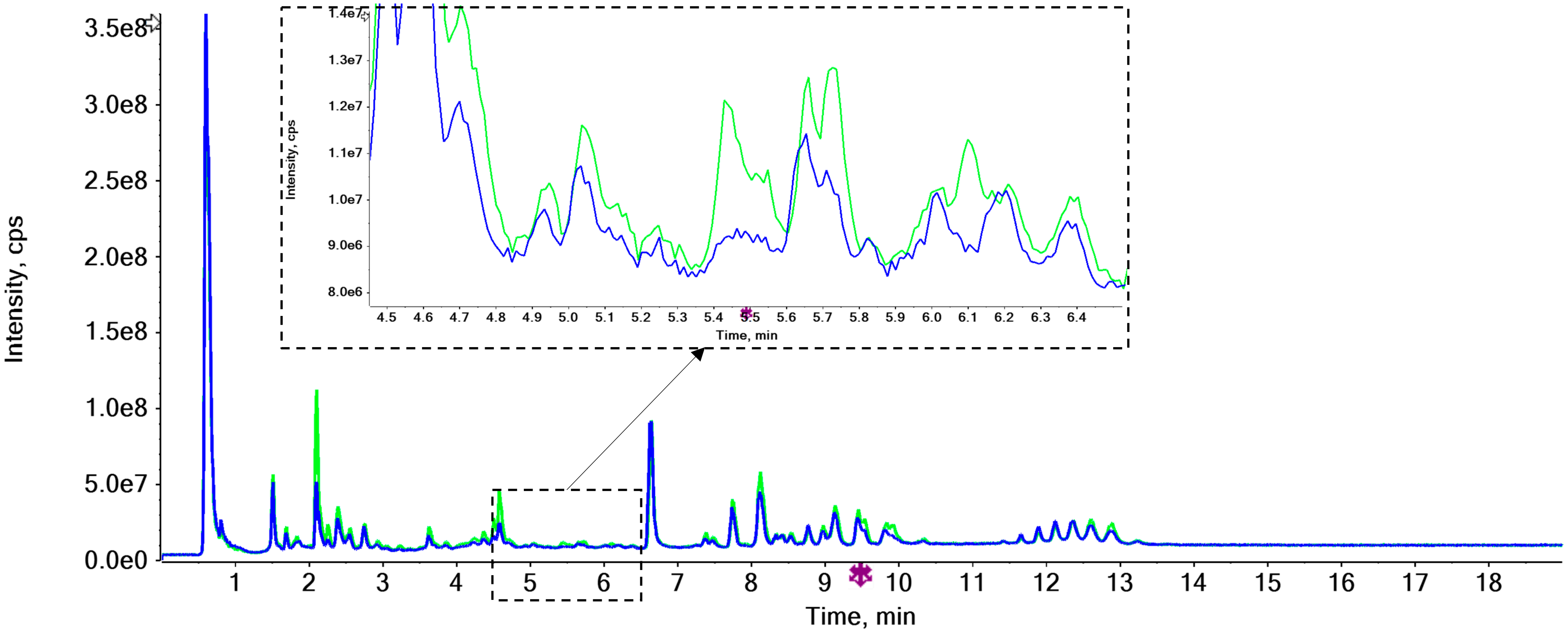

Figure S1. UHPLC-HRMS fingerprints of PEF-treated (blue) and control (green) *F. culmorum* (FC) samples; MeOH extracts, ESI+. The zoomed-in areas of the TIC chromatograms highlight the differences in the low-intensity regions.

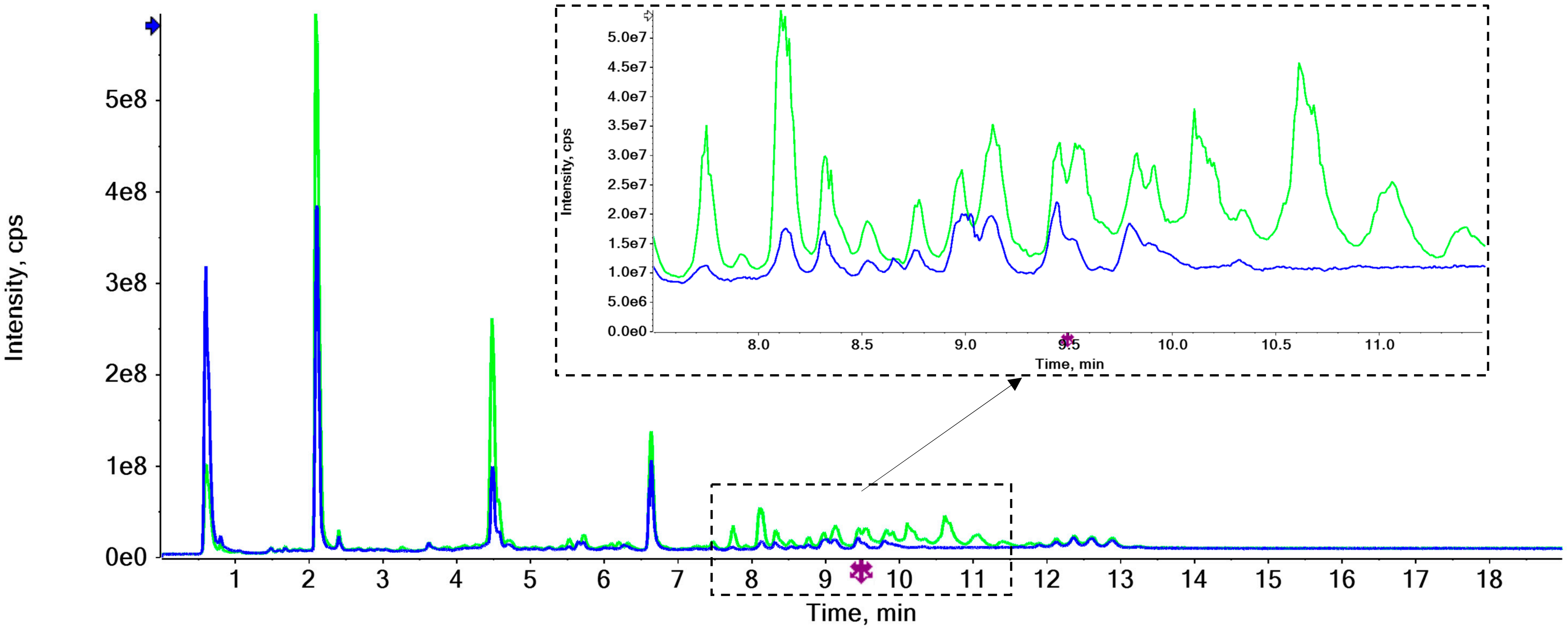

Figure S2. UHPLC-HRMS fingerprints of PEF-treated (blue) and control (green) *F. graminearum* (FG) samples; MeOH extracts, ESI+. The zoomed-in areas of the TIC chromatograms highlight the differences in the low-intensity regions.

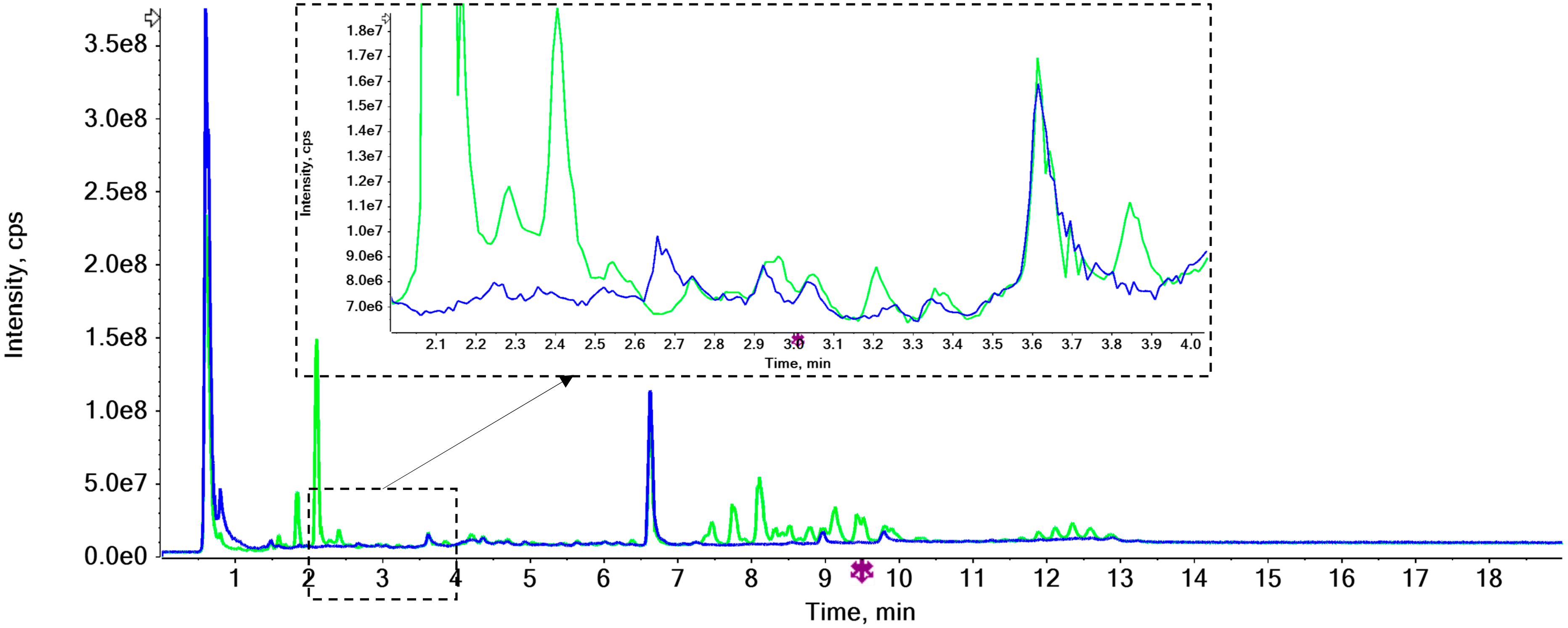

Figure S3. UHPLC-HRMS fingerprints of PEF-treated (blue) and control (green) *F. poae* (FP) samples; MeOH extracts, ESI+. The zoomed-in areas of the TIC chromatograms highlight the differences in the low-intensity regions.

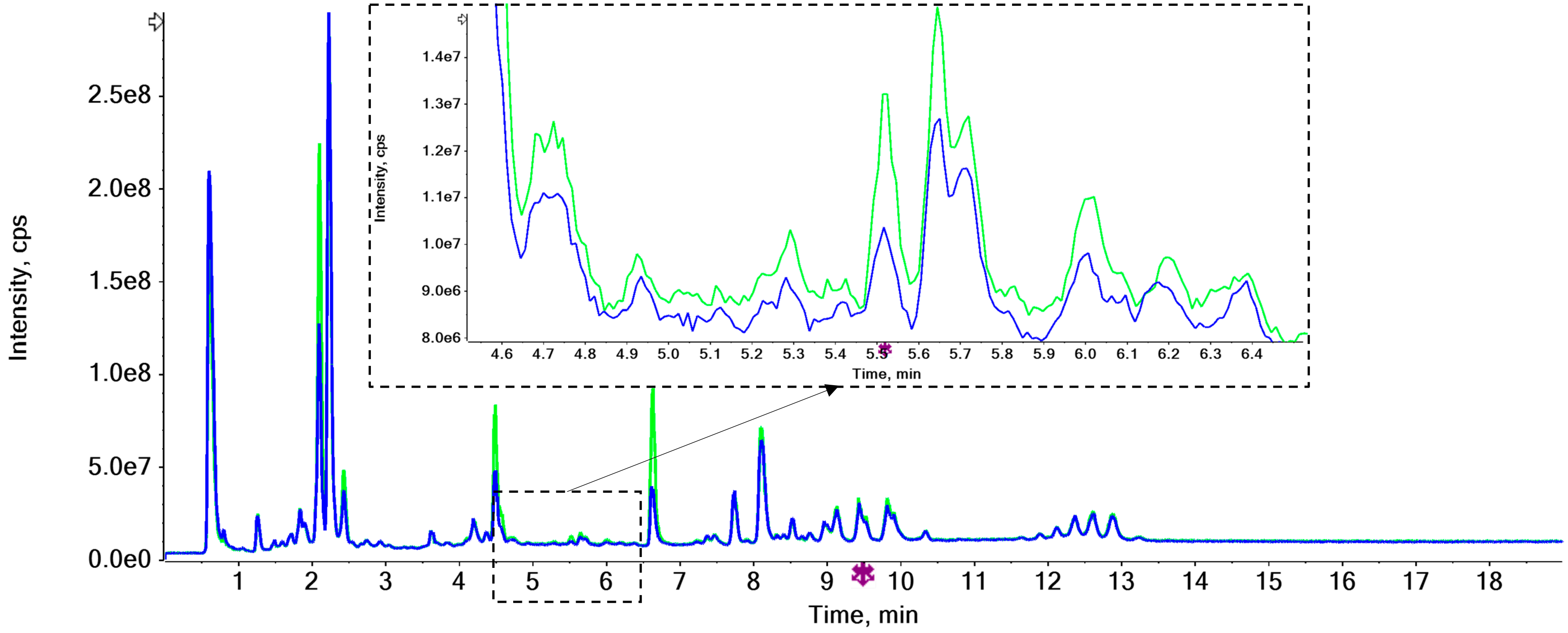

Figure S4. UHPLC-HRMS fingerprints of PEF-treated (blue) and control (green) *F. sporotrichioides* (FS) samples; MeOH extracts, ESI+. The zoomed-in areas of the TIC chromatograms highlight the differences in the low-intensity regions.

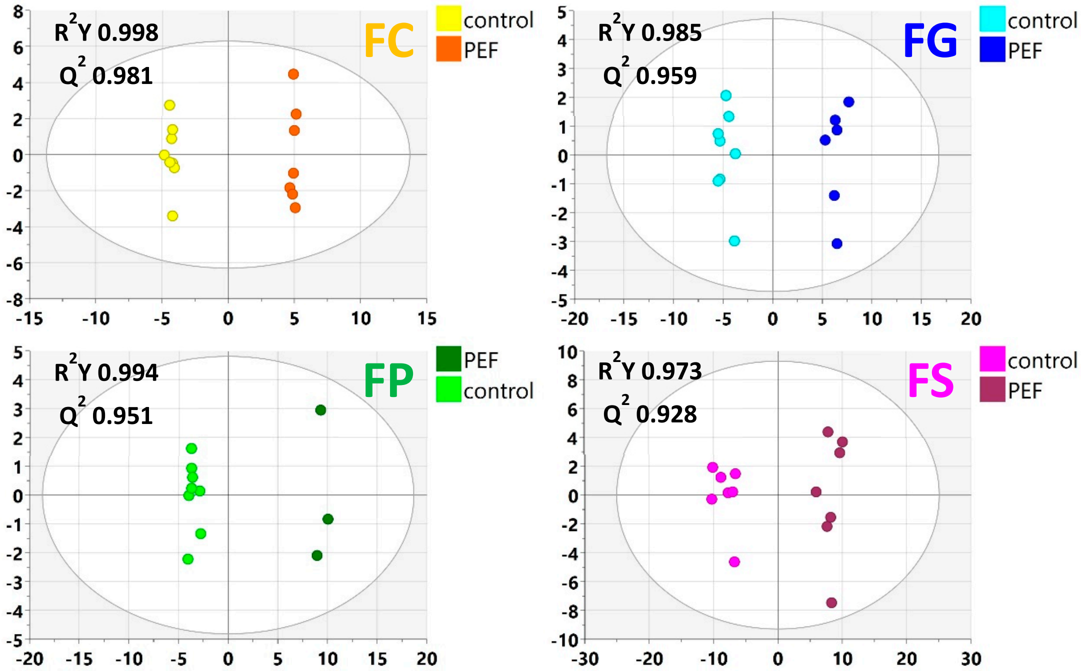

Figure S5. The OPLS-DA models (score scatter plot) of each *Fusarium* species dataset (FC—*F. culmorum*; FG—*F. graminearum*; FP—*F. poae*; FS—*F. sporotrichioides*) colored according to PEF treatment (PEF) and control with excellent values of quality parameters.
